# Formability and Failure Evaluation of AA3003-H18 Sheets in Single-Point Incremental Forming Process through the Design of Experiments

**DOI:** 10.3390/ma14040808

**Published:** 2021-02-08

**Authors:** Mohanraj Murugesan, Dong Won Jung

**Affiliations:** Department of Mechanical Engineering, Jeju National University, Jeju-Do 63243, Korea; mohanaero32@jejunu.ac.kr

**Keywords:** single incremental sheet forming, design of experiment, grey relational analysis, ANOVA, microstructure, response surface methodology

## Abstract

The single-point incremental forming process (SPIF) is one of the emerging manufacturing methods because of its flexibility in producing the desired complex shapes with higher formability at low-cost compared to traditional sheet forming methods. In this research work, we experimentally investigate the forming process to determine the influence of process parameters and their contribution to enhancing the formability without causing a fracture by combining the design of experiments (DOE), grey relational analysis (GRA), and statistical analysis of variance (ANOVA). The surface morphology and the energy dispersive X-ray spectroscopy (EDS) method are used to perform elemental analysis and examine the formed parts during three forming stages. The DOE procedure, a central composite design with a face-centered option, is devised for AA3003-H18 Al alloy sheet for modeling the real-time experiments. The response surface methodology (RSM) approach is adopted to optimize the forming parameters and recognize the optimal test conditions. The statistically developed model is found to have agree with the test measurements. The prediction model’s capability in $R^{2}$ is computed as 0.8931, indicating that the fitted regression model adequately aligns with the estimated grey relational grade (GRG) data. Other statistical parameters, such as root mean square error (RMSE) and average absolute relative error (AARE), are estimated as 0.0196 and 2.78%, respectively, proving the proposed regression model’s overall closeness to the measured data. In addition, the prediction error range is identified as −0.05 to 0.05, which is significantly lower and the residual data are distributed normally in the design space with variance and mean of 3.3748 and −0.1232, respectively. ANOVA is performed to understand the adequacy of the proposed model and the influence of the input factors on the response variable. The model parameters, including step size, feed rate, interaction effect of tool radius and step size, favorably influence the response variable. The model terms *X*_2_ (0.020 and 11.30), *X*_3_ (0.018 and 12.16), and *X*_1_*X*_2_ (0.026 and 9.72) are significant in terms of *p*-value and F-value, respectively. The microstructural inspection shows that the thinning behavior tends to be higher as forming depth advances to its maximum; the deformation is uniform and homogeneous under the predefined test conditions.

## 1. Introduction

**Figure 1:**
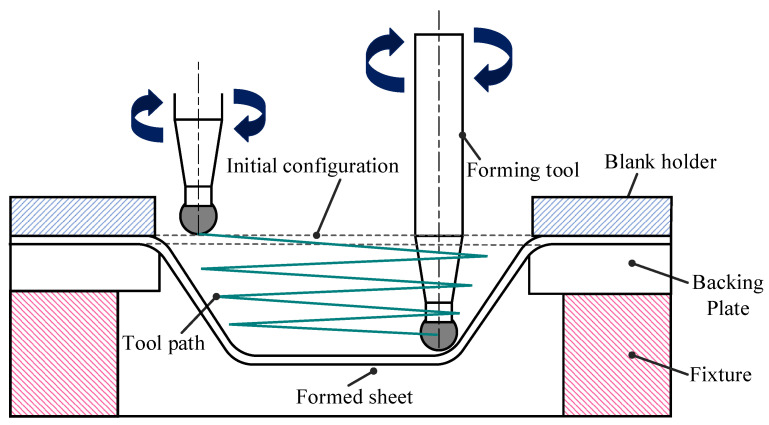
Schematic diagram of the negative incremental forming process.

Aluminum alloys continue to be widely used in industrial applications such as automobile components, aircraft structures, and ship panels because of their excellent mechanical properties like ductility, corrosion resistance, fracture toughness, light weight, and low cost [1,2]. Nowadays, manufacturing procedures need to be optimized to reduce the production cost and time without compromising the product quality. Existing conventional metal forming methods are designed to produce only predefined shapes; in the case of design alteration, the entire experimental setup has to be redesigned in terms of manufacturing tools. This kind of preparation requires more production time, increasing the costs by manufacturing new parts such as dies, punches, and molds [3]. However, the incremental sheet forming process (ISF) does not require any external die to produce the desired components as the new parts can be manufactured using the predefined contour tool path. This process uses the designed tool path to create a step-by-step deformation on the sheet metal part using the punch tool; a conceptual schematic diagram of the SPIF process is illustrated in Figure 1. However, the dimensional accuracy of the manufactured product from the SPIF process mainly depends on the working parameters such as punch tool radius, vertical step size, lubrication, spindle speed, and material selection and design parameters like geometry shape, sheet thickness, and wall angle [4,5,6,7,8,9]. The forming force also has a significant impact in the ISF process because excessive forming force can tear the sheet material due to thinning behavior, and the moderate forming force cannot deform the sheet metal to the desired shape [10,11,12]. So, choosing the proper forming punch tool is critical for preventing fractures and producing flawless parts [13,14,15].

Saidi et al. [16] investigated the process considering two materials, titanium grade 2 and AISI 304L stainless steel, to obtain the optimum forming force to ensure that both parts and the machine are adequately controlled to eliminate the failures. Three parameters (maximum tensile strength, tool diameter, and tool displacement) were adopted to develop a mathematical model for minimizing the forming force. For that purpose, a RSM, along with the Taguchi method, was used. They concluded that the considered forming parameters significant influenced the formed parts. Bao et al. [17] proposed an electro-pulse-assisted incremental forming process. The experimental results for AZ31B alloy material showed that the forming limit angle increased and the material formability improved because of the peak current density. From the microstructure analysis, they found that the material dynamic recrystallization (DRX) temperature decreased due to the electro pulse, accelerating the DRX progress and controling the crack growth in the tested material. Baruah et al. [18] aimed to improve formability and minimize surface roughness in the ISF process. The grey relational analysis (GRA) procedures considering Taguchi’s method were adopted, and surface roughness measurement from three directions— rolling, transverse, and angular—was used as the response variable. They reported that the formability contribution was mainly based on the lubrication, and conversely, the feed rate had the least contribution. Maqbool et al. [19] analyzed the relationship between dimensional accuracy and residual stresses considering various process parameters like tool diameter, tool step-down, and wall angle. From the detailed investigation, the wall angle was found to be the most significant working parameter, and the effects occurred mostly in the transverse direction of the punch tool movement. Ebot et al. [20] investigated the multi-pass single-point incremental forming process (MSPIF) process using an intermediate geometries tool-path strategy to improve the material formability. They provided the information for calculating the rigid body motion of the forming tool along the tool path. They concluded that using the proposed methodology, an MSPIF approach, improves material formability.

The tool path design strategies have been proven to significantly affect the forming process and alter the material formability, resulting in loss of achievable part accuracy. So, choosing the proper optimal tool path and its sequences is vital for ensuring higher part accuracy. Amar et al. examined the problem mentioned above [21,22]. They [21] proposed an error prediction tool for generating an error response surface to continuously monitor the geometry deviations using the multivariate adaptive regression splines method. From the outcome, they found that the accuracy of the manufactured part improved significantly in terms of average absolute deviation when the developed response surfaces were combined with a rib offset strategy. In addition, the same authors [22] proposed a network analysis methodology to obtain various aspects that affect the final geometry accuracy using conceptual graphs and then the optimized contour paths achieved from the modeled framework. They concluded that the deviations were reduced adequately in the complete formed part.

In the SPIF process, understanding deformation and fracture mechanisms plays a significant role in enhancing the formability of the formed products; detailed investigations have been conducted by Said et al. [23], Mohammad et al. [24], and Shakir Gatea et al. [25]. Ben et al. [23] examined the damage mechanism in the SPIF process using numerical simulations considering the conical geometry. The user subroutine was developed, including two damage models: an elastoplastic model with quadratic yield criteria of Hill’48 and the mixed isotropic/kinematic hardening behavior for modeling the process. They found that a mixed formulation, including isotropic-kinematic hardening, more accurately captured the damage evolution. The phenomenological modified Mohr–Coulomb (MMC3) model was implemented into the user subroutine material card for the commercial numerical code to examine the ductile damage in SPIF by Mirnia et al. [24]. Using an inverse approach, the MMC3 criterion was evaluated using the devised tensile tests for AA6061-T6 aluminum alloy sheet, and localized deformations were examined in detail. They reported that a deviation of 10% was recorded with the experimental measurements, and the prediction of fracture locations agreed well with the real observations. A modified Gurson–Tvergaard-Needleman (GTN) damage model considering shear proposed by Gatea et al. [25] showed a better ductile damage prediction in the SPIF process compared to the original GTN model under shear loading conditions. They mentioned that in the forming process, the damage propagation tends to be accelerated because of the shear under meridional tensile stress. Memicoglu et al. [26] researched the importance of numerical modeling in the flexible forming process, as the forming process mechanism has not been completely studied. They developed fast simulation models by reducing the computational time up to 24 times for the SPIF process, and the maximum model shape error was around 8%. They stated that the presented approach could be adopted to model the forming process with reasonable computational time.

Chang et al. [27] investigated the ISF process by considering the irregular thickness distribution induced by elastic deflection and plastic deformation. A detailed study on the surface roughness development was conducted to understand the forming mechanism; from this experience, analytical models to predict roughness were constructed. The proposed model was tested by adopting a conical shape against different materials, and the surface morphologies were analyzed. Jawale et al. [28] conducted similar research to investigate the reasons behind the surface roughness improvement. A polycrystalline copper sheet with a truncated conical shape and various lubricants was considered, and tests were conducted until fracture. The formed parts were investigated from the microstructural point of view; the authors observed a significant lubrication influence on the surface. The forming tool marks and the grain boundaries were identified as the reason for surface roughness increase. Kumar et al. [29] investigated the influence of process parameters on the surface quality. of the formed parts. For obtaining the optimum level of forming parameters, the Taguchi method was chosen with the help of the design of experiments and ANOVA. The tool diameter was found to influence the surface roughness, whereas the tool shape and the lubricant also produced significant influence in terms of the surface roughness. The proposed prediction model results showed better agreement with the confirming experiments for the optimized parameters. Even though numerous research works have been completed, the SPIF process is still being developed to produce better components with good surface quality and formability [30,31,32,33].

In this work, process parameters, such as forming tool radius, step size, and feed rate, were selected to investigate the formability of AA3003-H18 Al alloy sheets to obtain optimum forming conditions to identify the important parameters that influence the forming process by applying the design of experiments (DOE) statistical approach and grey relational analysis (GRA). The lubricant, a combination of oil and grease, was chosen over other lubricants for the tests based on the measured average surface roughness from incrementally formed parts produced using various lubricants. Real-time experiments were conducted using the experimental design developed from the central composite design with a face-centered option. Response surface methodology (RSM) was adopted for developing a grey relational grade (GRG) prediction model against the forming parameters, and statistical analysis of variance (ANOVA) was used to identify the influence of the process parameters on the response variable. The proposed prediction model was reviewed using graphical and numerical validations. In addition, the elemental composition and distribution of the selected material were analyzed using an EDS setup, and the microstructure was inspected using field emission scanning electron microscopy (FESEM) to identify the thinning behavior and the deformation mechanism under the predefined test conditions.

## 2. Experimental Procedures

**Figure 2:**
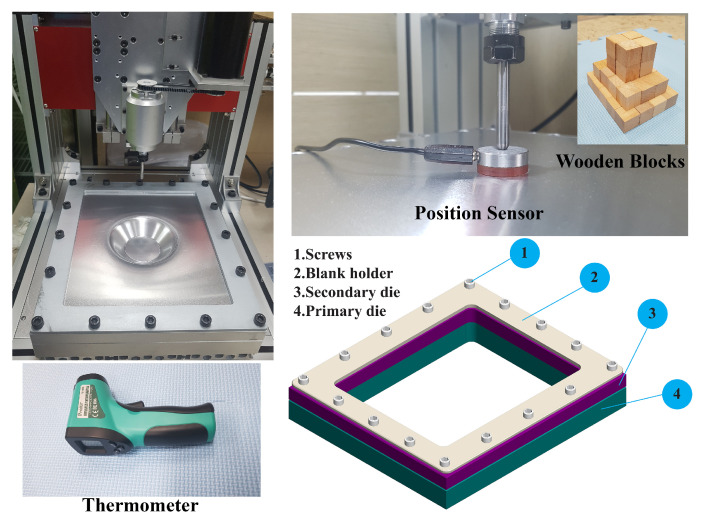
Experimental procedures of the single-point incremental forming process.

This section discusses the procedures used to carry out the SPIF process experiments in detail. The computer numerical control (CNC) machine used in this research work was custom-made to conduct the forming process and to investigate and understand the deformation mechanism behind the ISF process. Figure 2 shows the detailed experimental set-up of the SPIF process; as illustrated, it consisted of a few essential tools such as a position sensor, a specifically shaped forming tool, blank holder, die systems, holding screws, and a thermometer. The material sheet dimensions were chosen based on the working area of the customized design, and the chosen rectangular area of the aluminum sheet was 240×280 mm${}^{2}$ with a thickness of 0.5 mm. The forming tool was manufactured from the high-speed steel (HSS) material due to its material properties such as high hardness, wear resistance, and heat resistance. The typical chemical composition of the selected commercial aluminum alloy (AA3003) material are summarized in Table 1.

**Figure 3:**
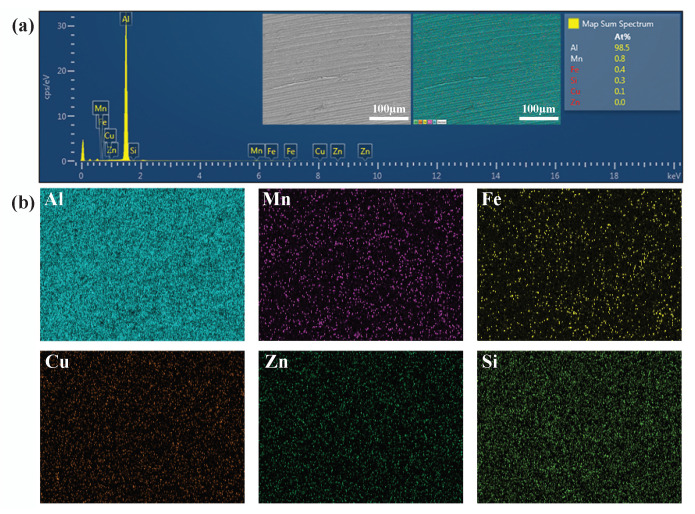
EDS analysis (**a**) element spectrum corresponding to AA3003. Inset images show the SEM-back scattered electron (BSE) scan area that was used for chemical composition analysis; (**b**) EDS element mapping images.

To confirm the presence of the alloy elements in the test samples, the elemental composition and distribution of the AA3003 material were determined using an EDS (MIRA3 TESCAN, secondary electron detector, Jeju National University, Jeju-si, South Korea) set-up at the magnification scale of 50 μm. The scanned surface distribution confirmed the existence of Al, Mn, Fe, Cu, Zn, and Si through the element spectrum results, as shown in Figure 3a. The EDS mapping surfaces showed that the scanned surface mainly contained Al; Cu was found to be lower in percentage and there was no Zn, as illustrated in Figure 3b. From the element spectrum results comparison, the presence of alloy elements was found to be almost same as the standardized data from the material database, as outlined in Table 1.

**Figure 4:**
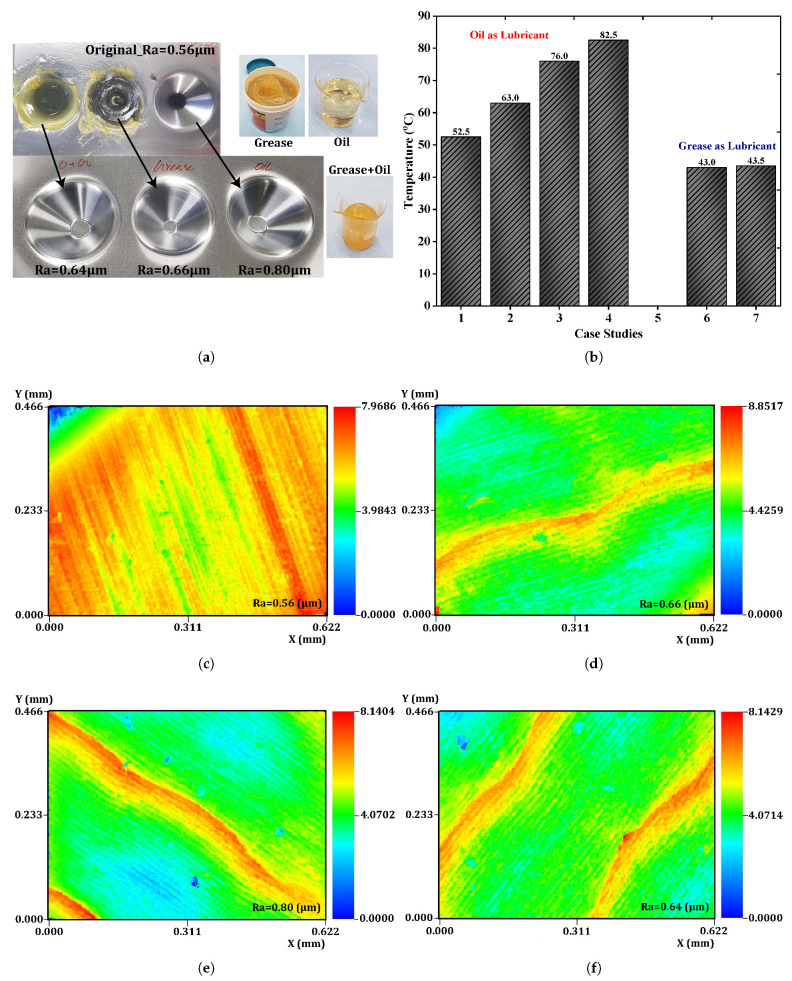
(**a**) Assessment of lubricants; (**b**) temperature comparison plot; 2D surface roughness profile of the formed material using different lubricants from the 3D nano profiler: (**c**) original surface; (**d**) grease; (**e**) oil; (**f**) combination of oil and grease.

To quantitatively evaluate the effect of lubricant performance on the surface quality assessment, the prepared sample was investigated using three different lubricants: oil, grease, and a combination of oil and grease, as displayed in Figure 4a. The forming conditions were modeled to be the same for all tested cases to facilitate comparison for choosing the best lubricant. Using 3D nano surface profiler equipment (Jeju National University, Jeju-si, South Korea), the roughness was measured as 0.56, 0.80, 0.66, and 0.64 μm for the original surface, oil lubricant, grease lubricant, and oil–grease lubricant, respectively. From the outcome, the lubricant combining oil and grease was found to be the best option. The scanned 2D surface roughness profile of the samples is illustrated in Figure 4c–f.

The contact during the forming process between the blank and the generally shaped forming tool is quick, transient, and temporary, which poses challenges for measuring the temperature changes in the test sample on the exact contact-forming area. However, in this research, the temperature changes were monitored and recorded from the lubricant using a manually controlled thermometer at a specified machining time to provide the results of heat transfer from the work piece and the forming tool to the lubricant during the forming process. Figure 4b shows that the oil lubricant tended to produce higher temperature changes than the grease lubricant because of its semisolid state and viscosity. As viscosity mainly depends on the temperature, the heat transfer among the grease lubricant and the test piece caused the viscosity of the grease to lower, increasing its temperature to a certain point, as shown in Figure 4a (grease turned black). The temperature of oil–grease lubricant did not change during the forming process, and the lubricant had a lower roughness, which was more favorable for producing a better surface quality on the formed parts compared to the other lubricants.

## 3. Microstructure Evaluation of the Formed Product

**Figure 5:**
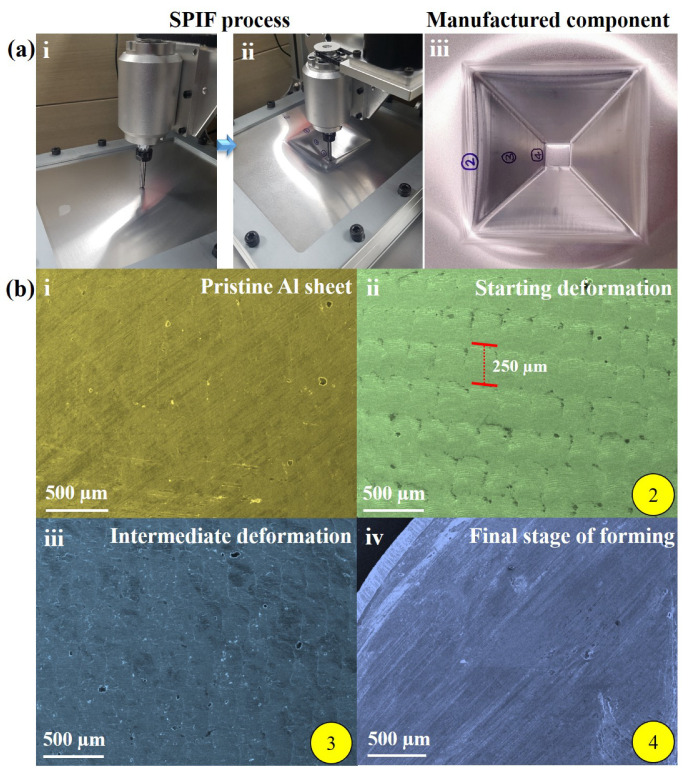
(**a**) SPIF process illustration and final part; (**b**) microstructure of aluminum alloy (AA3003) material at various forming stages, observation by FESEM at 500 μm magnification.

The field emission scanning electron microscopy (FESEM) (MIRA3 TESCAN, Secondary electron detector, Jeju National University, Jeju-si, South Korea) set-up was used to investigate the surface morphology. Stretching behavior occurs during the single-point incremental forming (SPIF) process, producing pyramid geometry, as shown in Figure 5a. Here, the stretching or elongation of the AA3003 material sheet during the SPIF process depended on the various working factors such as tool radius ( 3 mm), feed rate ( 1000 mm/min), forming depth ( 40 mm), spindle speed (5000 rpm), and step-size ( 0.25 mm). Figure 5a(iii) presents the desired pyramid-shaped product using AA3003 material, showing multiple regions; the flat (pristine and marked as 4), bending, and stretching locations (marked as 2 and 3, respectively) confirmed the variations in the punch tool path, friction, and material ductility.

During the initial state, the punch tool did not interact with the AA3003 sheet and the corresponding surface morphology was smooth: no stretched or elongated regions were observed, as shown in Figure 5b(i). A similar surface morphology was observed during the final stage of the SPIF process of creating pyramid geometry, as shown in Figure 5b(iv). However, in the case of regions 2 and 3, different types of periodic stretching regions formed on the AA3003 sheet due to the defined step-size ( 250 μm) of the punch tool movements in the SPIF process to create the pyramid shape, as shown in Figure 5b(ii,iii). Here, the magnification scale of all the FESEM images is 500 μm and regions 2 and 3 are interesting due to the differences in the nature of the stretching/bending (or thinning locations) of the AA3003 sheet.

**Figure 6:**
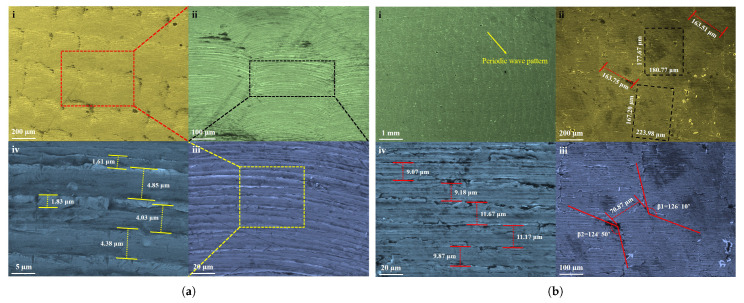
Microstructure observation of aluminum alloy (AA3003) material by FESEM at various magnifications at: (**a**) location 2; (**b**) location 3.

Figure 6a(i–iv) shows that the initial stretching of the AA3003 sheet is similar to the woven shaped morphology with a uniform step size and periodic nature. In Figure 6a(ii), the marked location shows the formation of multiple stackings of aluminum strips, and the magnified images (Figure 6a(iii,iv)) depict the linear arrangement of the strips, having an average width of 4.42 and 1.72 μm, respectively. Figure 6b(i–iv) shows that the surface morphology of the intermediate deformation of the pyramid sheet produced through the SPIF process is similar to the periodic wavy pattern shape constructed by the stackings of the multiple rectangular boxes, having a uniform area. The magnified image demonstrates the length, width, and order of the interfacial region between the rectangular box, as shown in Figure 6b(ii). The deformation of the aluminum sheet with respect to the angle (≈ 125 ${}^{\circ }$) and distance ( 70.87 μm) between the rectangular boxes similarly confirms the deformation process was homogeneous, as shown in Figure 6b(ii,iii). The rectangular box iformed by the periodic arrangement of linear strips with an average width of 10.19 μm, as shown in Figure 6b(iv). Here, there was more stretching (or thinning) of aluminum compared to the deformation of region 2 (Figure 6b(iv)). However, no failure conditions were experienced during the SPIF process of forming the pyramid geometry at the pre-defined forming depth and testing conditions.

## 4. Surrogate Modeling

### 4.1. Response Surface Methodology

Response surface methodology (RSM) is a statistical tool that is useful for developing and analyzing the relationship between the independent and the dependent parameters of the individually designed problem. This method uses an appropriate selection of the design of experiments (DOE) method to construct a suitable empirical mathematical model through the appropriate fitting of the real-time test data and discussion of the interaction effects of he input variables against the output variables. The second-order polynomial model, which contains both linear and non-linear terms along with interaction terms, used for proposing the mathematical model with 3 regressor variables, *k*, can be expressed as [34,35]:

(1) $$y_{i}=a_{0}+a_{1}x_{i1}+a_{2}x_{i2}+a_{3}x_{i3}+a_{4}x_{i1}^{2}+a_{5}x_{i2}^{2}+a_{6}x_{i3}^{2}+a_{7}x_{i1}x_{i2}+a_{8}x_{i2}x_{i3}+a_{9}x_{i3}x_{i1}+\epsilon ,$$

where *i*=1, 2,…, *n*. Assuming $x_{i4}$ = $x_{i1}^{2}$, $x_{i5}$ = $x_{i2}^{2}$, $x_{i6}$ = $x_{i3}^{2}$, $x_{i7}$ = $x_{i1}x_{i2}$, $x_{i8}$ = $x_{i2}x_{i3}$, and $x_{i9}$ = $x_{i3}x_{i1}$, Equation (1) can be rewritten in a simplified form as [34,35]:

(2) $$y_{i}=a_{0}+\sum_{i=1}^{n}\sum_{j=1}^{p}a_{j}x_{ij}+\epsilon .$$

In Equation (2), $a_{0}$ and $a_{j}$ are the regression coefficients, *n* is the total number of test points, and *p*, (k×3), is a limit calculated based on the number of independent variables (*k*).

Equation (2) can be expressed more conveniently using matrix notation:

y=Xa+ϵ,

where y(n×1), X(n×p), a(p×1), and ϵ(n×1) are the response observations, the independent variables, the regression coefficients, and the noise measurements [34,35] in vector and matrix forms, respectively. Through error minimization between the test and predicted samples, the model regression coefficients can be estimated using the least-squares procedures. Therefore, the least-squares estimate of *a* is [34,35]:

$$\hat{a}={\left(XX\right)}^{-1}Xy.$$

Eventually, from the estimated model coefficients, $\hat{a}$, the responses, $\hat{y}$, at unknown samples can be calculated as [34,35]:

$$\hat{y}=X\hat{a}.$$

### 4.2. Design of Experiments

**Figure 7:**
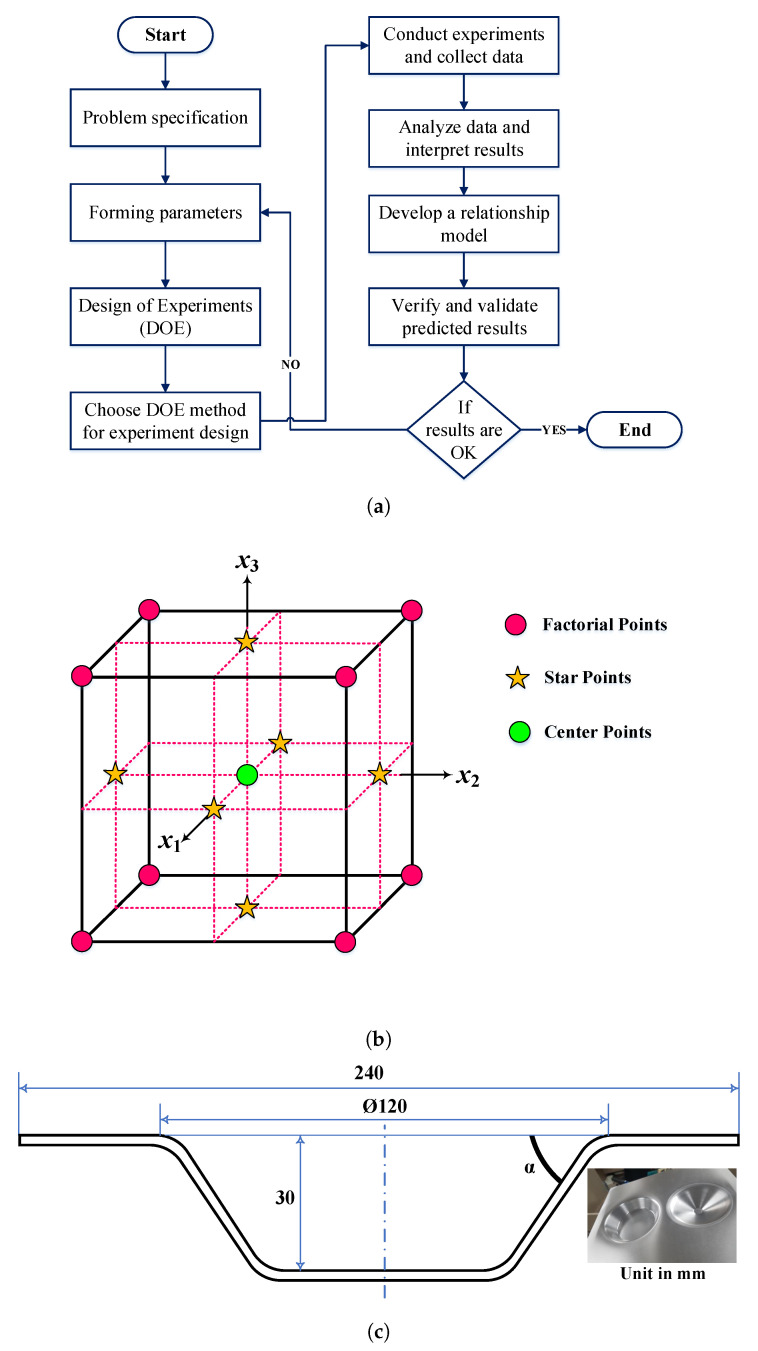
(**a**) Flow chart of experimental design process; (**b**) CCF design; (**c**) truncated conical geometry.

The DOE is well-suited for evaluating the input variables that significantly influence the response variables outcome in the design space. This tool is robust as it can be exploited in any experimental situation to weigh and examine the factors that control other working parameters. For determining the important missing interaction terms that increase the possibility of capturing the desired output, the input factors can be altered and investigated simultaneously using the DOE approach [34]. The detailed procedures to conductc the DOE process are illustrated in Figure 7a, and the main steps are classified as planning, conducting, analyzing, and interpreting the outcome of the real experiments. In this research, the real-time SPIF experiments were conducted using the modified CNC vertical milling machine. For conducting the experiments, the experimental design was applied using statistical software Minitab 18, and the experimental design was a 3-factor, 3-level factorial experiment, which are referred to as low, medium, and high levels. The forming parameters, including tool radius, step size, and feed rate, were customized according to the capability of the designed CNC machine, and Table 2 outlines the control forming parameters chosen for the experiments and their design spaces with various levels.

**Figure 8:**
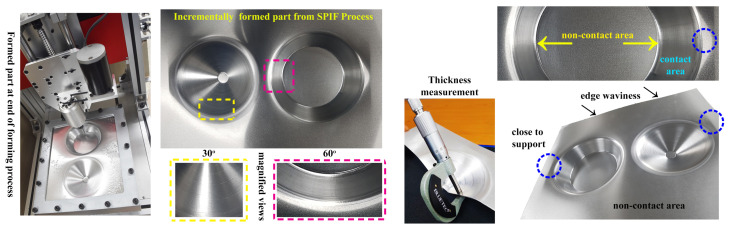
Incrementally formed truncated conical shape parts and thickness measurement.

**Figure 9:**
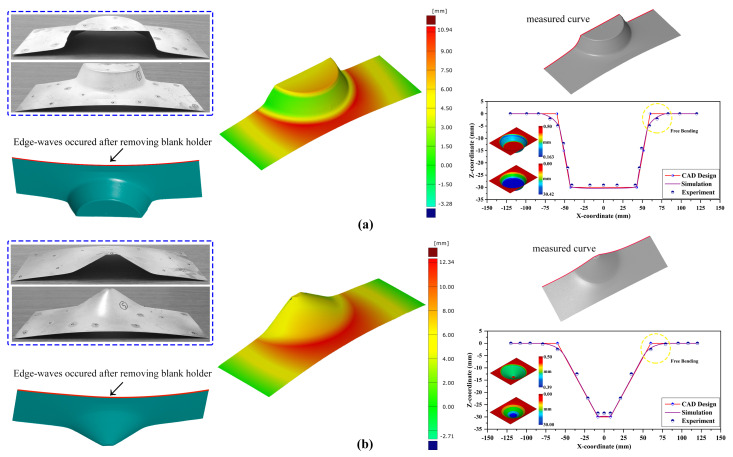
Estimated shape error using the ATOS 3D scanner. (**a**) 60°; (**b**) 30°.

Per the central composite design (CCD) considering the face-centered option (Figure 7b), the 20 sets of the experimental runs were obtained as factorial points (1–8), star points (9–14), and center points (15–20), as summarized in Table 3 and Table 4. Because of the 6 similar center points concerning the central composite face-centered (CCF) design, the responses were averaged from the repetition of the SPIF experiments without altering any test conditions. After each experiment (Figure 7c), the tested material surface roughness (Ra) was measured from three different locations [36], and the average value of the surface roughness was taken, as summarized in Table 3 and Table 4. The measurement procedures were repeated for the other output responses including thickness, forming time, angle, and height, and we obtained fifteen sets of test results, as documented in Table 3 and Table 4. The procedures used to measure the thickness in the formed part using a micrometer screw gauge are presented in Figure 8. To determine the shape error in terms of free bending and edge waviness, the incrementally produced parts were approximately cut down the middle; then, the prepared samples were checked for fractures and used for 3D scanning to obtain the cross-section coordinates and to estimate the shape error. For the 3D scanning process, the cut-down samples were prepared with a minimum of 12 reference point targets using 3D scan mark dot stickers. Here, the ATOS 3D Scanner was used for the three-dimensional measurements of the formed parts, as shown in Figure 9. The main advantage of this scanner is that it does not need any physical probes to manually touch multiple coordinates. The scanning process was repeated multiple times to improve the model accuracy, and the scanned model was stored in a computer-aided design (CAD) file.

### 4.3. Grey Relational Analysis

Grey relational analysis (GRA) is employed to determine the best combination of input parameters by converting a multi-objective problem into a single-objective problem to achieve the most reliable response for the chosen output parameters. This method is widely implemented to evaluate and assess the performance of a selected complex problem or a problem with multiple output responses. For obtaining accurate solutions, a particular set of sequences, as outlined in the algorithm table alg:thealg, has to be performed using the test data acquired from the real-time experiments for the chosen response variables. Firstly, the experimental observations were normalized using operation 1 for the chosen response variables, as summarized in Table 5, called grey relational generations. The sheet thickness, the formed angle, and height were assumed to be the-higher-the-better among the selected variables. In contrast, the machining time and surface roughness were considered to better the smaller the values. The deviation sequence, which is the absolute value of the difference between $x_{0j}\left(k\right)$ and $x_{ij}\left(k\right)$, was calculated (Table 6) using operation 2. Considering the distinguishing coefficient, ζ, as 0.5, the grey relational coefficient was estimated using operation 3, as tabulated in Table 7. Before performing operations 4 and 5, the weight estimation was required for the selected responses; the Shannon entropy method, widely adopted in the decision-making process, was used for the estimation process [37,38,39,40,41,42,43].

(3) $$p_{ij}=\frac{x_{ij}}{\sum_{i=1}^{m}x_{ij}}⟶E_{ij}=-k\sum_{i=1}^{m}p_{ij}\ln p_{ij}\;\;\;\;\mathrm{in}\;\mathrm{which}\;\;k={}^{1}\;{/}_{\ln(m)}$$

(4) $$w_{j}=\frac{1-E_{j}}{\sum_{j=1}^{n}\left(1-E_{j}\right)}⟶w_{j}^{*}=\frac{s_{j}w_{j}}{\sum_{j=1}^{n}s_{j}w_{j}}$$

At first, the response variables normalized to achieve the design outcomes ($p_{ij}$) and then the entropy measures computation of the design outcomes ($E_{ij}$) were estimated using Equation (3). Likely from Equation (4), the standard form of entropy weight estimation ($w_{j}^{*}$) was established by combining the subjective ($s_{j}$) and objective weights ($w_{j}$); the weights of the parameters were determined to be $w_{1}$=$w_{2}$=$w_{3}$=$w_{4}$=$w_{5}$=0.2, which means an equal weight for each response. Then, the grey relational grade (GRG) was computed using the weights estimated from the Shannon entropy weighting method and operations, 4 and 5, as summarized in Table 8. The optimal combination was determined from the higher GRG rank, which was experimental run 14, as listed in Table 8.
**Algorithm 1:** Procedures used for grey relational analysis (GRA) [37,38,39,40,41,42,43]._1_Normalization: If the likelihood is the-smaller-the-netter (SB) or the-higher-the-better (HB),
$$\mathrm{SB}:\;\;x_{ij}\left(k\right)=\frac{\max\;x_{ij}\left(k\right)-x_{ij}\left(k\right)}{\max\;x_{ij}\left(k\right)-\min\;x_{ij}\left(k\right)}\;\;\;\;\mathrm{HB}:\;\;x_{ij}\left(k\right)=\frac{x_{ij}\left(k\right)-\min\;x_{ij}\left(k\right)}{\max\;x_{ij}\left(k\right)-\min\;x_{ij}\left(k\right)}$$_2_Evaluation of $\Delta_{ij}$ : $\Delta_{ij}=|x_{0j}\left(k\right)-x_{ij}\left(k\right)|$_3_Grey relational coefficient calculation:$$\gamma \left(x_{0j},x_{ij}\right)=\frac{\Delta_{\min}+\zeta \Delta_{\max}}{\Delta_{ij}\left(k\right)+\zeta \Delta_{\max}}⟵\Delta_{ij}=|x_{0j}\left(k\right)-x_{ij}\left(k\right)|,$$
where $\gamma (x_{0j},x_{ij})$ is the grey relational coefficient between $x_{ij}$ and $x_{0j}$. $\Delta_{\max}$ is the maximum value of $\Delta_{ij}$, and $\Delta_{\min}$ is the minimum value of $\Delta_{ij}$. ζ is the distinguishing coefficient (0≤ζ≥1), and assumed to be 0.5._4_From the grey relational coefficient, the grey relational grade (GRG) is determined as follows:$$\gamma_{i}=\frac{1}{n}\sum_{i=1}^{n}\gamma \left(x_{0j},x_{ij}\right).$$_5_Considering the weighting method in real-time applications, the GRG can be rewritten as:$$\gamma_{i}=\frac{1}{n}\sum_{i=1}^{n}w_{k}\gamma \left(x_{0j},x_{ij}\right),$$
where $w_{k}$ is the weighting factor for *k*. In the present investigation, the weighting value $w_{k}$ for the response each parameter was estimated from the Shannon entropy weighting method._6_Rank according to the values of the GRG in decreasing order.

### 4.4. Numerical Verifications for Checking the Regression Model

Mathematical assessments such as the coefficient of determination ($R^{2}$), adjusted $R^{2}$, root mean square error (RMSE), and average absolute relative error (AARE) are widely used for verifying the adequacy of the proposed regression model [44,45,46,47].

#### 4.4.1. Coefficient of Determination ($R^{2}$)

The most common parameter used to determine a regression model’s adequacy is $R^{2}$, which is the square of the correlation coefficient between the actual and the predicted data. This parameter indicates the association between any two quantitative variables by explaining the amount of variation appearing in the data; the greater the value, the higher the quality of fit [44,45,46,47].

(5) $$R^{2}=1-\frac{SS_{E}}{SS_{T}}$$

where $SS_{E}$=$\sum_{i=1}^{n}{\left(y_{e}^{i}-y_{p}^{i}\right)}^{2}$ and $SS_{T}$=$\sum_{i=1}^{n}{\left(y_{e}^{i}-{\bar{y}}_{e}\right)}^{2}$; $SS_{E}$ and $SS_{T}$ are the error sum of squares and the total corrected sum of squares, respectively. In Equation (5), $y_{e}$, $y_{p}$, and ${\bar{y}}_{e}$ are the test data, the predicted data, and the averaged experimental observations, respectively.

#### 4.4.2. Adjusted $R^{2}$

Adjusted $R^{2}$ is the altered form of $R^{2}$, which has been modified based on the number of independent variables in the regression model. This quantity is always smaller than $R^{2}$ because it improves only when a new regressor variable is included in the regression model [44,45,46,47].

(6) $$\mathrm{Adjusted}\;R^{2}=1-\frac{{}^{SS_{E}}\;{/}_{\left(n-K-1\right)}}{{}^{SS_{T}}\;{/}_{\left(n-1\right)}}$$

#### 4.4.3. Root Mean Square Error (RMSE)

RMSE reveals how the residuals are presented in the designed space. It is often used to estimate the difference between the actual and the predicted data; e.g., if RMSE is near to 0, the calculated data are scattered adjacent to the regression line, and vice versa [44,45,46,47].

(7) $$\mathrm{RMSE}=\sqrt{\frac{\sum_{i=1}^{n}{\left(\mathrm{test}\;{\mathrm{data}}_{i}-\mathrm{predicted}\;{\mathrm{data}}_{i}\right)}^{2}}{n}}$$

#### 4.4.4. Average Absolute Relative Error (AARE)

AARE is the same as the RMSE metric, except the prediction error from the proposed model against the test observations is determined from term-by-term data comparison [44,45,46,47].

(8) $$\mathrm{AARE}=\frac{1}{n}\sum_{i=1}^{n}\left|\frac{\mathrm{test}\;{\mathrm{data}}_{i}-\mathrm{predicted}\;{\mathrm{data}}_{i}}{\mathrm{test}\;{\mathrm{data}}_{i}}\right|\times 100\%.$$

### 4.5. Results and Discussion

This section discusses the surface finish of the incrementally formed parts, the influence of the process parameters on the response variable from ANOVA results, the shape error of the produced parts using 3D scanning, and the microstructure evaluation of the perfectly formed parts and fractured samples using SEM analysis. At first, we aimed to form a part with a 60 mm outer radius, 30 mm forming height, and two forming angles (30° and 60°) using the SPIF process, as shown in Figure 8. For this investigation, the influences of the forming parameters, including tool radius, vertical step–size, and feed–rate, were considered, whereas other parameters like sheet thickness, tool shape, and lubricant remained constant. As an example, an incrementally formed part at the end of the forming process is depicted in Figure 8; the magnified views are provided to show the part’s surface finish. The magnified pictures show that the forming tool’s contour path can be noticed with the 30° truncated cone shape compared to the 60° cone shape. Edge waviness is noticed on the formed sheet at the fixture location, which occurred due to material flexibility and sheet fluctuations during the continuous incremental forming process. In detail, the fluctuations occurred during the forming process because the incremental forming process intends to form a material sheet gradually and locally by applying the punch force at a specified location; as the process continues based on the predefined tool path, the fluctuations tend to occur throughout the entire process. However, close to the support, as highlighted in Figure 8, this edge waviness was not witnessed due to the strong bending at that location.

The desired 3D CAD models were compared against the scanned models to check for the presence of deviations, as depicted in Figure 9. As shown in Figure 9a,b, the formed parts tended to have an extra backward bending at the fixture location in both forming angle cases. Apart from the test results, the 3D scanned contour shows that deviations were identified as 10.94 mm for 60° and 12.34 mm for 30° truncated conical geometries. The slightest error may have occurred in the scanned model data due to the reference point alignment during model comparison. However, the data were cross-verified by manual measurement, and the computed shape deviation was confirmed to be close to the manual estimation. For evaluating the accuracy of the formed part, the finite element model was modeled, and the mechanical properties achieved from the tensile test were incorporated into the material card, MAT18, which can consider the power law of plasticity for describing the material’s plastic behavior. The FEM accuracy was controlled with a reasonable number of elements in the blank material and was considered to be deformable; the forming tool was meshed with coarse elements and considered to be rigid. Due to the lubrication selection, a combination of oil and grease, the friction coefficient was assumed to be almost close to zero. To reduce the computation time, the blank sheet mesh was considered to be a shell element with five integration points, whereas the punch tool was also assumed to be a shell element with the same number of integration points. As shown in Figure 9a,b, the cross-section coordinates were compared among the CAD data, test data, and numerical simulation data. We confirmed that the models had free edge bending close to the start of the forming location, as magnified in the comparison plots, due to lack of extra support at the location and the material adaptability to punch force. The free bending can be observed in detail in the scanned contours (Figure 9a,b, red color regions close to initial forming location). The same procedures were followed for the other tested samples, and the data of final thickness, forming height, forming angle, and average surface roughness were collected for performing the statistical analysis to build a regression model of grey relational grade.

(9) $$\begin{array}{c} \mathrm{GRG}=0.6399+0.0004\;x_{1}+0.0356\;x_{2}+0.0369\;x_{3}-0.0146\;x_{1}^{2}-0.0463\;x_{2}^{2}\\ +0.0190\;x_{3}^{2}+0.0369\;x_{1}x_{2}+0.0129\;x_{2}x_{3}-0.0078\;x_{3}x_{1} \end{array}$$

**Figure 10:**
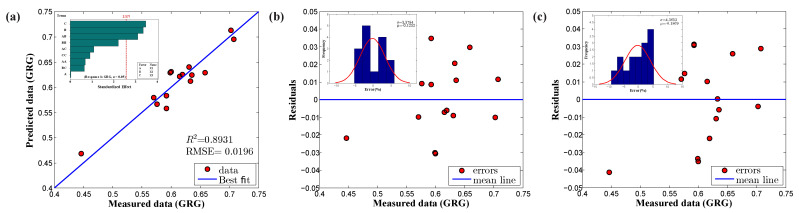
Full model (**a**) correlation plot; (**b**) residual plot; (**c**) residual plot of reduced model.

The multilinear regression model with linear, square, and interaction effects was chosen for developing the prediction model of GRG values using Table 8. The obtained prediction model with the model coefficients is presented in Equation (9). The achieved regression model was further numerically validated. Statistical metrics for verification were assessed and are compiled in Table 9. The prediction model’s capability in $R^{2}$ was computed as 0.8931, indicating that the fitted regression model adequately aligned with the estimated GRG data. An $R^{2}$ value of 0.8931 indicated that the input parameter’s variance explained 89.31% of the response variable’s variance. As shown in Table 9, the adjusted–RSQ values were quite close to $R^{2}$, but slightly less, typically occurring as the number of input factors increases. Other statistical parameters, such as RMSE and AARE, were estimated as 0.0196 and 2.78%, respectively, proving the overall closeness of the proposed regression model to the measured data. Apart from the numerical metrics, graphical verification was performed, as shown in Figure 10a,b. The relationship plot (Figure 10a) provides visual information about how the predicted data were distributed around the best fit line; we clearly observed that the predicted samples laid very close to the fitted regression line. The residual plot was constructed based on the differences between the measured and predicted data, as illustrated in Figure 10b. Figure 10b shows that the residual population followed the random pattern, which proved the usefulness of the proposed empirical model; the residual data fell close to the zero error line, except for a few outliers. However, the error range was identified to be between −0.05 and 0.05, which is p significantly lower. The histogram plot of the residuals was constructed (Figure 10b, inset image) and we found that the residual data were distributed normally in the design space with variance and mean of 3.3748 and −0.1232, respectively.

Analysis of variance (ANOVA) results for the proposed prediction model are listed in Table 9. The proposed model F-value was found to be 4.64 (GRG), indicating that the developed model is statistically significant overall in terms of the process parameters: feed rate, vertical step-size, and tool radius. A *p*-value less than 0.05 indicates the importance of the forming parameters that influence the response variable (the grey relational grade.) The model terms $X_{2}$ (0.020), $X_{3}$ (0.018), and $X_{1}X_{2}$ (0.026) were found to be significant as they had *p*-values less than 0.05, while the other terms were found to be insignificant. Besides the *p*-value, another statistical parameter, the F-value, was found to be 11.30 ($X_{2}$), 12.16 ($X_{3}$), and 9.72 ($X_{1}X_{2}$), showing the importance of the computed model terms. The proposed model contributed 89.29% to capturing the response variable in the design space, while the model error was computed to be 10.7%. In detail, the model terms $X_{2}$, $X_{3}$, $X_{2}X_{2}$, and $X_{1}X_{2}$ contributed 24.18%, 26.02%, 10.50%, and 20.80%, respectively, more than the other model terms.

**Figure 11:**
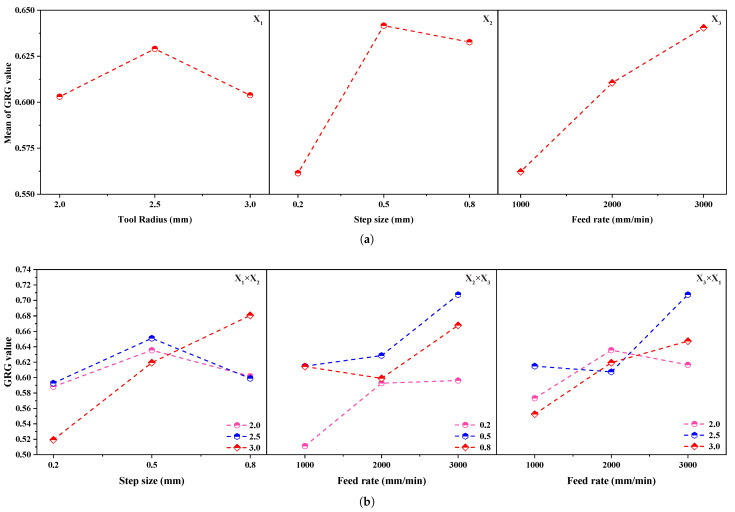
Process parameters vs. GRG values: (**a**) main effects; (**b**) interaction effects.

To examine the influence of the input parameters on the response, the average response of each level input factor is calculated and plotted using the line graph. If the estimated data points form a horizontal line, then there is no main effect. Conversely, if the plotted data points represent positive or negative deflection to the horizontal line, then the main effect is presented in the regression model [48]. To investigate the main effect of the input factors on the grey relational grade, the graphical illustration of the proposed regression model, i.e., 2D line plot, was used, as shown in Figure 11a, and the computed GRG values are presented in Table 10. Figure 11a depicts that the model terms $X_{1}$ (tool radius) and $X_{2}$ (step–size) provided a combination of positive and negative responses on the output variable. In detail, the GRG value increased with the tool radius, $X_{1}$, up to 2.5 mm, and then decreased to 3.0 mm; similarly, it increased linearly with the step size, $X_{2}$, up to 0.5 mm, and then dropped to 0.8 mm. However, the model term $X_{3}$ (feed rate) showed an entirely positive response and was identified as the most significant parameter, whereas the model term $X_{2}$ (step size) was chosen as the second most important parameter that influenced the response. Table 10 provides the ranking information about the input parameter’s influences on the response.

The interaction plots are used here to represent how the relationship between one process parameter and a response variable, the mean GRG value, depends on the second process parameter value. The computed GRG values are summarized in Table 11. For example, Figure 11b displays the mean GRG for the levels of one process parameter on the *x*-axis and a separate line for each level of another process parameter. The plotted lines in Figure 11b were evaluated in detail to explain and understand how the interactions affect the relationship between the input factors and the response (mean GRG value). The plotted lines can be interpreted using two options: parallel lines, which mean there is no interaction between input factors and response; and nonparallel lines, in which an interaction occurs among the input factors and response. For example, if more nonparallel lines are identified, then the interaction strength is more significant. In this interaction plot (Figure 11b), the lines are not parallel for the model terms $X_{1}$ and $X_{2}$. This interaction effect indicated that the relationship between step size and mean GRG value depended on the tool radius value. For example, if a step–size of 0.8 mm is considered, a tool radius of 3.0 mm is associated with the highest mean GRG value. Conversely, in Figure 11b, the lines are almost parallel for the model terms $X_{2}$, $X_{3}$, and $X_{3}X_{1}$, indicating no relationship among them on the response variable. As the interaction effects were significant in the model term $X_{1}X_{2}$, the main effects cannot be interpreted without acknowledging the interaction effects of the $X_{1}$ and $X_{2}$ factors. Even though the plots represent the interaction effect, other statistical parameters in the ANOVA test have to be evaluated to confirm the effect’s statistical significance. From the ANOVA results in Table 9, the developed regression model’s F-value, and *p*-value of interaction term $X_{1}\times X_{2}$ are 9.72 (GRG) and 0.026 (GRG) (<0.05), respectively, showing that the interaction effect of $X_{1}\times X_{2}$ on the mean GRG value is statistically significant.

(10) $$\mathrm{GRG}=0.6416+0.03561\;x_{2}+0.03693\;x_{3}-0.0445\;x_{2}^{2}+0.0369\;x_{1}x_{2}$$

Model reduction is one of the regression model strategies that allow the user to simplify a developed regression model by omitting insignificant model terms using ANOVA results. The advantage of model reduction is that terms reduction can make the model easier to working with; notably, sometimes, omitting insignificant terms can decrease model accuracy. In this work, Equation (9) was reduced by manually eliminating insignificant model terms (*p*-value > 0.05), and the remaining model terms (*p*–value < 0.05 and 0.10 [49]) were used to build a new regression model. The reduced model is presented in Equation (10). From Equation (10) and Table 12, the model terms are perceived to be statistically significant based on each coefficient’s *p*-value; however, the statistical parameters $R^{2}$, adjusted–$R^{2}$, and RMSE showed trivial results in terms of model prediction. Besides, the proposed model was observed to be insignificant through graphical verification, such as residual and histogram plots, as the residuals were distributed randomly with too many outliers, as shown in Figure 10c. Eventually, the response optimizer tool was used to identify the combination of input factor settings that optimize the response (GRG value). From the optimizer, the optimal solution of the response variable, a GRG value of 0.7081, was identified using the optimum forming parameters: 3.0 mm tool radius, 0.745 mm vertical step size, and 3000 mm/min feed rate.

**Figure 12:**
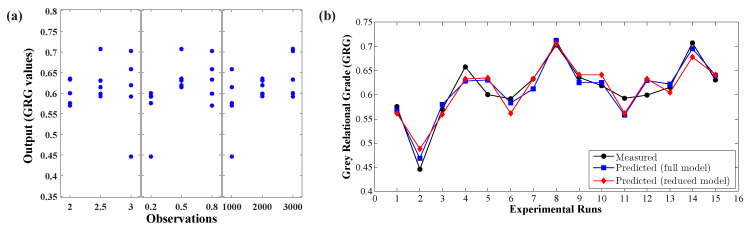
(**a**) Scatter plot of observed data; (**b**) measured vs. predicted data of GRG value.

From Figure 12a, the sample’s distribution shows that these observations were obtained from a three-level design and shows linearity. However, it reveals that the deviations occurred in the model prediction for the tested input points due to the presence of outliers; the data deviations at each level follow vertical data distribution. To represent the proposed regression model’s usefulness, the measured data, GRG values, were compared against the predicted data between the full and reduced empirical models, as shown in Figure 12b. The observation revealed that the predicted data fell close to the measured data (GRG values) for most observations. The reduced regression model displayed a considerable deviation in the prediction response. Overall, the statistical approach exhibited here can be used to predict the GRG values or any selected single objective for the preferred material to produce parts with better formability, surface finish, and faster machining time by thoroughly reviewing the process parameters.

**Figure 13:**
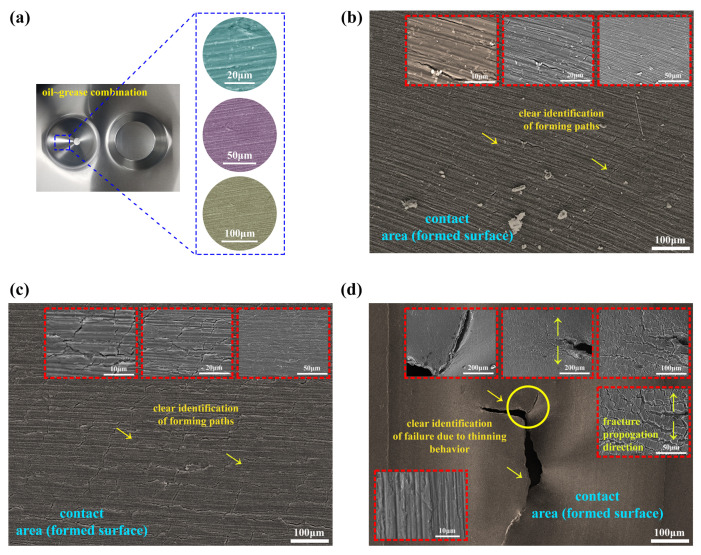
FESEM images at various magnifications: (**a**) surface from oil–grease lubricant used; (**b**) unfractured scanned surface from 60° part; (**c**) unfractured scanned surface from 30° part; (**d**) fractured surface.

The incrementally produced part’s external surface (tool contact region) using a lubrricant combining oil and grease was examined using FESEM pictures, as depicted in Figure 13a. Figure 13a, depicting the magnified scanning electron microscope (SEM) images at 20, 50, and 100 μm, shows that the surface tended to have a uniform deformation during the forming process; because of this observation, the smooth transition between the forming tool and the blank was presumed. Figure 13b,c illustrates the scanned surfaces of the formed samples from the fourth experimental design; Table 3 and Table 4 provide results at magnification levels of 10, 20, 50, and 100 μm. Figure 13b shows the apparent association of forming paths due to the punch tool movement along the blank with the predefined vertical step size for 60° truncated conical shape geometry. As the magnification levels increased (Figure 13b), at 10 and 20 μm, the insets show that a uniform region was observed to have irregular micro-cracks between the forming path lines. This response occurred due to the thinning that occurs during the forming process; if more thinning occurs, it might lead to a fracture in the test sample. Similarly, in Figure 13c, the same kind of consistent homogeneous deformation response is observed in the scanned surface at 100 μm by identifying forming paths. However, the scanned surface tended to have more irregular micro-cracks all over the surface, and the vertical step size was usually responsible for these micro-cracks observations. The insets in Figure 13c depict the scanned surface’s amplified images at 10 and 20 μm; from the inset images, micro-cracks can be seen more clearly. Notably, the formed part did not have any visual cracks; the cracks were only observed in FESEM photographs. However, the fractured sample from thinning failure occurrence was cut down for examining the outer (tool contact region) surface. The magnification of a fractured surface at the 100 μm scale is depicted in Figure 13d. The SEM pictures of a fractured surface at higher magnifications in various regions are illustrated in Figure 13d (insets); the insets show that the fracture was predominantly ductile. As shown in the insets in Figure 13d at the 50 and 100 μm scales, the damage concentrated due to nucleation, then slowly evolved as voids coalesced, and propagated into near regions as secondary voids coalescence. This continuous nucleation of tiny voids coalescence led to the fracture during the forming process. In conclusion, FESEM investigations are useful for understanding and observing the deformation mechanisms behind the SPIF process.

## 5. Conclusions

In this research, we experimentally investigated the SPIF process to explore the influence of the process parameters and their contribution to improving the formability without causing a fracture by combining DOE, GRA, and ANOVA. For performing the SPIF process, the lubricant, a combination of oil and grease, was found to be most suitable based on surface roughness measurements from a 3D nano surface profiler compared to other lubricants (oil and grease alone). From the element spectrum results, the presence of alloy elements was found to be almost same as the standardized data from the material database. The effect of forming process parameters in the grey relational grade was investigated using response surface methodology, adopting a central composite design with a face-centered option to model the real-time experiments. Overall, the proposed regression models were verified using numerical and graphical validations. The statistically proposed regression model was found to agree with the experimental measurements, having a higher correlation coefficient and lower prediction error. The prediction model’s capability in RSQ was computed as 0.8931, indicating that the fitted regression model adequately aligned with the estimated GRG data. Other statistical parameters, RMSE and AARE, were estimated as 0.0196 and 2.78%, respectively, proving the proposed regression model’s overall fit with the measured data. In addition, the prediction error range was identified as being between −0.05 and 0.05, which is significantly lower, and the residual data were recognized as having randomness in the residuals population and the normal distribution in the design space, with variance and mean of 3.3748 and −0.1232, respectively. ANOVA was performed to understand the adequacy of the proposed model and the influence of the input factors on the response variable. The model parameters (step size, feed rate, interaction effect of tool radius, and step size) were found to favorably influence the response variable. The model terms $X_{2}$ (0.020 and 11.30), $X_{3}$ (0.018 and 12.16), and $X_{1}X_{2}$ (0.026 and 9.72) were found to be significant in terms of *p*-value and F-value, respectively. The microstructural inspection showed that the thinning behavior tended to increase as forming depth reached its maximum; the deformation was also seen to be uniform and homogeneous under the predefined test conditions. The statistical approach presented here can be used as a guideline to understand the forming process; it will also be useful for performing the SPIF process to improve product formability for any selected material. 

## Figures and Tables

**Table 1:** Chemical composition of aluminum alloy (AA3003) material.

Composition	Al	Si	Fe	Cu	Mn	Zn
wt %	96.8–99.0	0.6	0.7	0.05–0.20	1.0–1.5	0.10

**Table 2:** Design spaces of forming parameters and their levels.

Variables		Levels		
		Low	Center	High
		−1	0	+1
Tool radius (mm)	$x_{1}$	2.0	2.5	3.0
Step size (mm)	$x_{2}$	0.2	0.5	0.8
Feed rate (mm/min)	$x_{3}$	1000	2000	3000

**Table 3:** CCF experimental design and experimental results using a 30° cone angle.

Wall Angle (θ), Time (*t*), Height (*h*), and Roughness (Ra)								
Run	$x_{1}$	$x_{2}$	$x_{3}$	Thickness (mm)	θ (${}^{\circ }$)	t (min.s)	h (mm)	Ra (μm)
1	2.0	0.2	1000	0.475	28.8	32.45	27.8	0.755
2	3.0	0.2	1000	0.460	29.0	32.30	28.0	1.690
3	2.0	0.8	1000	0.475	31.1	9.05	25.5	2.080
4	3.0	0.8	1000	0.485	30.0	9.01	28.6	1.070
5	2.0	0.2	3000	0.470	29.8	11.05	27.4	0.820
6	3.0	0.2	3000	0.460	29.2	11.00	29.6	1.290
7	2.0	0.8	3000	0.485	28.9	3.11	29.0	1.395
8	3.0	0.8	3000	0.470	29.0	3.10	27.3	0.880
9	2.0	0.5	2000	0.455	29.7	7.01	27.0	0.788
10	3.0	0.5	2000	0.470	29.8	6.58	27.6	1.190
11	2.5	0.2	2000	0.455	29.8	16.27	28.2	0.880
12	2.5	0.8	2000	0.480	29.8	4.39	26.5	1.500
13	2.5	0.5	1000	0.475	31.0	13.45	28.9	0.930
14	2.5	0.5	3000	0.475	30.0	4.45	28.5	1.140
15–20	2.5	0.5	2000	0.470	31.5	7.00	27.5	0.990

**Table 4:** CCF experimental design and experimental results using a 60° cone angle.

Wall Angle (θ), Time (*t*), Height (*h*), and Roughness (Ra)								
Run	$x_{1}$	$x_{2}$	$x_{3}$	Thickness (mm)	θ (${}^{\circ }$)	t (min.s)	h (mm)	Ra (μm)
1	2.0	0.2	1000	0.290	59.0	48.36	28.9	0.516
2	3.0	0.2	1000	0.295	57.7	48.04	28.6	1.090
3	2.0	0.8	1000	0.280	61.8	13.14	27.6	1.610
4	3.0	0.8	1000	0.295	58.2	13.06	29.6	1.310
5	2.0	0.2	3000	0.270	58.7	16.22	28.2	0.498
6	3.0	0.2	3000	0.270	60.0	16.11	29.7	1.463
7	2.0	0.8	3000	0.275	59.5	4.34	27.4	1.860
8	3.0	0.8	3000	0.320	61.5	4.32	27.8	1.060
9	2.0	0.5	2000	0.300	59.5	10.13	28.0	0.507
10	3.0	0.5	2000	0.320	57.2	10.07	28.7	1.010
11	2.5	0.2	2000	0.250	59.6	24.17	29.4	0.488
12	2.5	0.8	2000	0.315	59.2	6.42	25.9	1.410
13	2.5	0.5	1000	0.280	59.0	20.06	29.4	1.647
14	2.5	0.5	3000	0.270	62.0	6.52	29.8	1.230
15–20	2.5	0.5	2000	0.270	58.7	10.10	29.0	1.320

**Table 5:** Grey relational generation values.

	Wall Angle (30${}^{\circ }$)					Wall Angle (60${}^{\circ }$)				
**Run**	$y_{1}$ **(mm)**	$y_{2}$ **(${}^{\circ }$)**	$y_{3}$ **(min.s)**	$y_{4}$ **(mm)**	$y_{5}$ **(μm)**	$y_{1}$ **(mm)**	$y_{2}$ **(${}^{\circ }$)**	$y_{3}$ **(min.s)**	$y_{4}$ **(mm)**	$y_{5}$ **(μm)**
	**HB**	**HB**	**SB**	**HB**	**SB**	**HB**	**HB**	**SB**	**HB**	**SB**
1	0.667	0.000	0.000	0.561	1.000	0.571	0.375	0.000	0.769	0.980
2	0.167	0.074	0.005	0.610	0.294	0.643	0.104	0.007	0.692	0.561
3	0.667	0.852	0.797	0.000	0.000	0.429	0.958	0.800	0.436	0.182
4	1.000	0.444	0.799	0.756	0.762	0.643	0.208	0.802	0.949	0.401
5	0.500	0.370	0.729	0.463	0.951	0.286	0.313	0.730	0.590	0.993
6	0.167	0.148	0.731	1.000	0.596	0.286	0.583	0.732	0.974	0.289
7	1.000	0.037	1.000	0.854	0.517	0.357	0.479	1.000	0.385	0.000
8	0.500	0.074	1.000	0.432	0.906	1.000	0.896	1.000	0.487	0.583
9	0.000	0.333	0.867	0.366	0.975	0.714	0.479	0.868	0.538	0.986
10	0.500	0.370	0.881	0.512	0.672	1.000	0.000	0.869	0.718	0.620
11	0.000	0.370	0.551	0.659	0.906	0.000	0.500	0.549	0.897	1.000
12	0.833	0.370	0.956	0.244	0.438	0.929	0.417	0.952	0.000	0.328
13	0.667	0.815	0.647	0.829	0.868	0.429	0.375	0.643	0.897	0.155
14	0.667	0.444	0.954	0.732	0.709	0.286	1.000	0.950	1.000	0.459
15	0.500	1.000	0.867	0.488	0.823	0.286	0.313	0.869	0.795	0.394

**Table 6:** Evaluation of deviation sequence.

	Wall Angle (30${}^{\circ }$)					Wall Angle (60${}^{\circ }$)				
**Run**	$y_{1}$ **(mm)**	$y_{2}$ **(${}^{\circ }$)**	$y_{3}$ **(min.s)**	$y_{4}$ **(mm)**	$y_{5}$ **(μm)**	$y_{1}$ **(mm)**	$y_{2}$ **(${}^{\circ }$)**	$y_{3}$ **(min.s)**	$y_{4}$ **(mm)**	$y_{5}$ **(μm)**
	**HB**	**HB**	**SB**	**HB**	**SB**	**HB**	**HB**	**SB**	**HB**	**SB**
1	0.333	1.000	1.000	0.439	0.000	0.429	0.625	1.000	0.231	0.020
2	0.833	0.926	0.995	0.390	0.706	0.357	0.896	0.993	0.308	0.439
3	0.333	0.148	0.203	1.000	1.000	0.571	0.042	0.200	0.564	0.818
4	0.000	0.556	0.201	0.244	0.238	0.357	0.792	0.198	0.051	0.599
5	0.500	0.630	0.271	0.537	0.049	0.714	0.688	0.270	0.410	0.007
6	0.833	0.852	0.269	0.000	0.404	0.714	0.417	0.268	0.026	0.711
7	0.000	0.963	0.000	0.146	0.483	0.643	0.521	0.000	0.615	1.000
8	0.500	0.926	0.000	0.568	0.094	0.000	0.104	0.000	0.513	0.417
9	1.000	0.667	0.133	0.634	0.025	0.286	0.521	0.132	0.462	0.014
10	0.500	0.630	0.119	0.488	0.328	0.000	1.000	0.131	0.282	0.380
11	1.000	0.630	0.449	0.341	0.094	1.000	0.500	0.451	0.103	0.000
12	0.167	0.630	0.044	0.756	0.562	0.071	0.583	0.048	1.000	0.672
13	0.333	0.185	0.353	0.171	0.132	0.571	0.625	0.357	0.103	0.845
14	0.333	0.556	0.046	0.268	0.291	0.714	0.000	0.050	0.000	0.541
15	0.500	0.000	0.133	0.512	0.177	0.714	0.688	0.131	0.205	0.606

**Table 7:** Estimated values of the grey relational coefficient.

	Wall Angle (30${}^{\circ }$)					Wall Angle (60${}^{\circ }$)				
**Runs**	$y_{1}$ **(mm)**	$y_{2}$ **(${}^{\circ }$)**	$y_{3}$ **(min.s)**	$y_{4}$ **(mm)**	$y_{5}$ **(μm)**	$y_{1}$ **(mm)**	$y_{2}$ **(${}^{\circ }$)**	$y_{3}$ **(min.s)**	$y_{4}$ **(mm)**	$y_{5}$ **(μm)**
	**HB**	**HB**	**SB**	**HB**	**SB**	**HB**	**HB**	**SB**	**HB**	**SB**
1	0.600	0.333	0.333	0.532	1.000	0.538	0.444	0.333	0.684	0.961
2	0.375	0.351	0.334	0.562	0.415	0.583	0.358	0.335	0.619	0.533
3	0.600	0.771	0.712	0.333	0.333	0.467	0.923	0.714	0.470	0.379
4	1.000	0.474	0.713	0.672	0.678	0.583	0.387	0.716	0.907	0.455
5	0.500	0.443	0.649	0.482	0.911	0.412	0.421	0.649	0.549	0.986
6	0.375	0.370	0.650	1.000	0.553	0.412	0.545	0.651	0.951	0.413
7	1.000	0.342	0.999	0.774	0.509	0.438	0.490	0.999	0.448	0.333
8	0.500	0.351	1.000	0.468	0.841	1.000	0.828	1.000	0.494	0.545
9	0.333	0.429	0.790	0.441	0.953	0.636	0.490	0.791	0.520	0.973
10	0.500	0.443	0.808	0.506	0.604	1.000	0.333	0.793	0.639	0.568
11	0.333	0.443	0.527	0.594	0.841	0.333	0.500	0.526	0.830	1.000
12	0.750	0.443	0.919	0.398	0.471	0.875	0.462	0.913	0.333	0.427
13	0.600	0.730	0.586	0.745	0.791	0.467	0.444	0.583	0.830	0.372
14	0.600	0.474	0.916	0.651	0.632	0.412	1.000	0.909	1.000	0.480
15	0.500	1.000	0.790	0.494	0.738	0.412	0.421	0.792	0.709	0.452

**Table 8:** Computed values of the grey relational grade (GRG) and rank.

Run	1	2	3	4	5	6	7	8	9	10
**GRG**	0.576	0.446	0.570	0.658	0.600	0.592	0.633	0.703	0.636	0.619
**Rank**	13	15	14	3	9	12	5	2	4	7
**Run**	**11**	**12**	**13**	**14**	**15**					
**GRG**	0.593	0.599	0.615	0.707	0.631					
**Rank**	11	10	8	1	6					

**Table 9:** Analysis of variance (ANOVA) for GRG values.

Source	DF	Adj SS	Adj MS	F-Value	*p*-Value	Contribution (%)
Model	9	0.0468	0.0052	4.64	0.053	**89.299**
Linear Terms	3	0.0263	0.0088	7.82	0.025	50.212
X1	1	0.0000	0.0000	0.00	0.972	0.004
X2	1	0.0127	0.0127	11.30	**0.020**	24.188
X3	1	0.0136	0.0136	12.16	**0.018**	26.022
Square Terms	3	0.0078	0.0026	2.31	0.194	14.826
X1×X1	1	0.0006	0.0006	0.49	0.515	1.049
X2×X2	1	0.0055	0.0055	4.91	0.078	10.501
X3×X3	1	0.0009	0.0009	0.83	0.405	1.769
Interaction Terms	3	0.0127	0.0042	3.78	0.093	24.259
X1×X2	1	0.0109	0.0109	9.72	**0.026**	20.800
X1×X3	1	0.0013	0.0013	1.18	0.327	2.524
X2×X3	1	0.0005	0.0005	0.44	0.538	0.937
Error	5	0.0056	0.0011			**10.701**
Total	14	0.0524				
S = 0.0335		$R^{2}$ = 89.30%	Adjusted RSQ = 70.04%			

**Table 10:** Main effects of considered parameters on mean GRG values.

$X_{1}$	GRG	Rank	$X_{2}$	GRG	Rank	$X_{3}$	GRG	Rank
2.0	0.6030	3	0.2	0.5615	2	1000	0.5732	1
2.5	0.6290		0.5	0.6416		2000	0.6155	
3.0	0.6038		0.8	0.6327		3000	0.6417	

**Table 11:** Interaction effects of the selected parameters on mean GRG values.

$X_{1}X_{2}$	0.2	0.5	0.8	$X_{2}X_{3}$	1000	2000	3000	$X_{3}X_{1}$	2.0	2.5	3.0
2.0	0.5881	0.5927	0.5193	0.2	0.5112	0.6149	0.6144	1000	0.5732	0.6149	0.5525
2.5	0.6356	0.651	0.6194	0.5	0.5927	0.6286	0.599	2000	0.6356	0.6075	0.6194
3.0	0.6017	0.599	0.6806	0.8	0.5961	0.7074	0.6679	3000	0.6166	0.7074	0.6474

**Table 12:** Analysis of variance (ANOVA) for GRG values.

Source	DF	Adj.SS	Adj.MS	F-Value	*p*-Value	Contribution (%)
Model	4	0.0438	0.0110	12.76	0.001	**83.615**
X2	1	0.0127	0.0127	14.76	0.003	24.188
X3	1	0.0136	0.0136	15.88	0.003	26.022
Square	1	0.0066	0.0066	7.69	0.020	**12.605**
X2×X2	1	0.0066	0.0066	7.69	0.020	12.605
2-Way Interaction	1	0.0109	0.0109	12.69	0.005	**20.800**
X1×X2	1	0.0109	0.0109	12.69	0.005	20.800
Error	10	0.0086	0.0009			**16.385**
Total	14	0.0524				
S = 0.02931		$R^{2}$ = 83.62%		Adj.RSQ = 77.06%		

## Data Availability

The data presented in this study are available on request from the corresponding author.

## Abbreviations

The following abbreviations are used in this manuscript:

CNC	computer numerical control
ISF	incremental sheet forming process
SPIF	single-point incremental forming process
MMC3	modified Mohr–Coulomb
GTN	Gurson–Tvergaard–Needleman
HSS	high-speed steel
FE	finite element
DOE	design of experiments
CCD	central composite design
CCF	central composite face-centered
SEM	scanning electron microscope
FESEM	field emission scanning electron microscopy
EDS	energy-dispersive X-ray spectroscopy
$R^{2}$	coefficient of determination
RMSE	root mean square error
MSE	mean square error
AARE	average absolute relative error
RSM	response surface methodology
GRA	grey relational analysis
GRG	grey relational grade
ANOVA	statistical analysis of variance
CAD	computer-aided design
